# Predictive value of C-reactive protein/albumin ratio for no-reflow in patients with non-ST-elevation myocardial infarction

**DOI:** 10.34172/jcvtr.2022.30549

**Published:** 2022-11-22

**Authors:** Aydın Rodi Tosu, Tufan Çinar, Muhsin Kalyoncuoğlu, Halil İbrahim Biter, Sinem Çakal, Beytullah Çakal, Murat Selçuk, Erdal Belen, Mehmet Mustafa Can

**Affiliations:** ^1^Department of Cardiology, Haseki Training and Research Hospital, University of Health Sciences, Istanbul, Turkey; ^2^Department of Cardiology, Sultan II. Abdülhamid Han Training and Research Hospital, University of Health Sciences, Istanbul, Turkey; ^3^Department of Cardiology, Istanbul Medipol University, Faculty of Medicine, Istanbul, Turkey

**Keywords:** C-reactive Protein, Serum Albumin, No-Reflow, CAR

## Abstract

***Introduction:*** The focus of this research was to explore the link between CRP (C-reactive protein) /albumin ratio (CAR), a novel inflammatory response marker, and no-reflow (NR) phenomena in non-ST elevation myocardial infarction (non-STEMI) patients during percutaneous coronary intervention (PCI).

***Methods:*** The current study recruited 209 non-STEMI participants who underwent PCI. The patients were divided into two groups based on their post-intervention Thrombolysis in Myocardial Infarction (TIMI) flow grade; those with and without NR.

***Results:*** In all, 30 non-STEMI patients (6.9%) had NR after PCI. CAR values were substantially greater in the NR group. The CAR was identified to be a determinant of the NR (OR: 1.250, 95% CI: 1.033-1.513, *P*=0.02), although CRP and albumin were not independently related with NR in the multivariate analysis. In our investigation, low density lipoprotein-cholesterol levels and high thrombus burden were also predictors of the occurrence of NR. According to receiver operating characteristic curve evaluation, the optimal value of CAR was>1.4 with 60% sensitivity and 47% specificity in detecting NR in non-STEMI patients following PCI.

***Conclusion:*** To the best of knowledge, this is the first investigation to demonstrate that the CAR, a new and useful inflammatory marker, can be utilized as a predictor of NR in patients with non-STEMI prior to PCI.

## Introduction

 The no-reflow (NR) phenomenon is defined as a complicated situation characterized by insufficient coronary artery myocardial perfusion in the absence of angiographic epicardial vascular obstruction, spasm, or dissection.^1,2^ According to available data, individuals who had NR during both in primary and elective percutaneous coronary intervention (PCI) had death rates ranging from 2% to 44%, considerably greater than those who did not have NR.^3-5^ Although the NR phenomenon is not properly known, several explanations have been connected to it, including pre-existing microvascular impairment, distal microembolisation, ischemia damage, and hypoperfusion. Further, the secretion of numerous inflammatory markers in the context of an acute coronary syndrome (ACS) might exacerbate the inflammatory reaction and create oxygen free radicals, causing microvascular dysfunction and the NR event.^6,7^

 The C-reactive protein (CRP)/albumin ratio (CAR) has been recently developed as a potential inflammatory marker, and it is thought to better represent the outcomes in patients with several critical illnesses and malignancies than CRP and albumin alone.^8,9^ Importantly, in patients with ST elevation myocardial infarction (STEMI) having primary PCI, this index has been revealed to accurately predict in-hospital and long-term mortality, contrast-induced acute renal damage, and NR.^10-12^ Nonetheless, the predictive ability of CAR in identifying NR in non-ST elevation myocardial infarction (non-STEMI) patients having PCI has not been evaluated. Since NR was connected to the inflammatory condition and CAR was a stronger predictor of inflammation than CRP and albumin alone, the focus of this research was to explore the link between CAR and NR in non-STEMI patients during PCI.

## Material and Methods

###  Data collection

 The hospital data was used to obtain relevant information of consecutive non-STEMI patients who had coronary angiography (CAG) and PCI between January 2018 and December 2020. Individuals with chronic infection, chronic inflammatory conditions, decompensated heart failure, severe liver disease, active hepatitis, end-stage renal failure, cancer history, acute STEMI, and angina with normal cardiac biomarkers were excluded. Finally, the research included a total of 209 non-STEMI participants. Clinical and demographic features were recorded, including hypertension (HT), diabetes mellitus (DM), family history, hyperlipidemia, and smoking. The diagnosis of non-STEMI was consistent with the most current guideline.^13^ The Global Registry risk score for Acute Coronary Events (GRACE) was derived from the patient’s medical history, clinical symptoms, electrocardiography, and laboratory findings throughout hospitalization to assess the patient’s risk. Age, heart rate, systolic blood pressure, plasma creatinine, Killip class, ST segment deviation, elevated cardiac biomarkers, and cardiac arrest during admission constitute the GRACE risk scoring model. All participants in this research were managed in accordance with current guideline protocols.^13^ A fasting plasma glucose level of more than 125 mg/dL or the use of anti-diabetic medication was considered type 2 DM. HT was described as having a resting blood pressure greater than 140/90 mm Hg at least twice or being on antihypertensive drug treatment.

###  Laboratory measurements 

 Blood samples were drawn from the antecubital vein at 24 hours of hospitalization. An auto-analyzer was used to determine complete blood counts, including platelet, neutrophil, and lymphocyte counts. An automated chemistry analyzer was used to assess fasting blood glucose, total cholesterol, triglyceride, high-density lipoprotein-cholesterol (HDL-C), and low-density lipoprotein-cholesterol (LDL-C) in all patients. As in earlier investigations, CAR was determined as the ratio of serum CRP level to serum albumin level multiplied by 100 for ease of analysis.^10,14^

###  Coronary angiography and PCI

 Coronary scans were digitally captured for quantitative assessment (DICOM viewer; MedCom GmbH, Darmstadt, Germany). An urgent coronary angiography (CAG) was performed within 2 hours in very high-risk non-STEMI patients. In high-risk non-STEMI patients, CAG was conducted within 12 hours of hospital admission. Two competent interventional cardiologists who were anonymous to the patients’ clinical information reviewed all coronary scans. The anatomic severity of coronary stenosis was quantified using the anatomical Syntax Score I and II, which may be acquired at www.syntaxscore.com. According to the final thrombus burden, thrombus burden was classified as low (grades 1–3) or high (grades 4 and 5). After the PCI operation, the culprit vessel’s thrombolysis in myocardial infarction (TIMI) flow grade was assessed for post-procedural TIMI flow grade.^15^ NR was defined as TIMI flow grades 0 through 2 or TIMI flow grade 3 in combination with a final myocardial blush degree of less than two points, while reflow was defined as TIMI flow grade 3 in combination with a final myocardial blush degree of more than two points.^16^

###  Statistical analysis

 The Statistical Package for the Social Sciences version 24.0 (IBM Corp., Armonk, New York, USA) and MEDCALC (Software bvba 13, Ostend, Belgium) programs were used for data analysis. The continuous variables were expressed as means (standard deviations (SDs) (assuming normal distribution) and medians (interquartile ranges) (if not normal distribution). The percentages were assigned to the category variables. To analyze categorical data between groups, the Chi-squared (2) test was utilized. To determine if the variables were normally distributed or not, the Kolmogorov–Smirnov test was utilized. To examine continuous variables between groups based on whether they were uniformly distributed or not, the Student’s *t* test or Mann–Whitney U test was conducted. Univariate and multivariate logistic regression analysis (Backward LR technique) were used to obtain the independent determinants of NR. In the multivariate logistic regression analysis, only variables having a P value of less than 0.1 in the univariate analysis were included. CAR’s capacity to detect NR was determined using a receiver operating characteristic (ROC) curve study. From the point of maximum sensitivity and specificity (Youden’s index), the best cutoff value was obtained. The data were analyzed using a 95% confidence interval (CI) and a* P* value of < 0.05 as the statistical threshold.

## Results

 In total, 209 non-STEMI patients were included in the research, with the median age of 56 [48-65.6]. Males ratio was 79.4% (n: 166 patients) in overall cohort. In this analysis, 30 non-STEMI patients (6.9%) had NR after receiving PCI.

 According to the post-intervention TIMI flow grade, the study participants were divided into two groups: those with and without NR. Table 1outlines the demographic and clinical features of both groups. There was no difference in the proportion of comorbidities, such as HT, DM, or hyperlipidemia, across the groups. In addition, smoking status, family history, and coronary artery disease were all similar among groups. There was no difference between the groups in regard to medications. We found that both groups were comparable in regard to the GRACE score, in-hospital mortality, and cardiovascular-related hospitalization.

**Table 1:** Demographic and clinical parameters of the study cohort

	**All patients** **(n=209)**	**NR (-)** **(n=179)**	**NR (+)** **(n=30)**	***P *** **value**
Male gender, n (%)	166 (79.4)	143 (79.9)	23 (76.7)	0.69
Age, years	56 [48-65.6]	56 [48-66]	56 [48.5-63]	0.87
BMI, kg/m^2^	27 ± 3.0	26 ± 2.9	27 ± 3.7	0.18
Hypertension, n (%)	112 (52.6)	99 (55.3)	13 (43.3)	0.22
Diabetes mellitus, n (%)	59 (28.2)	50 (27.9)	9 (30)	0.82
Hyperlipidemia, n (%)	44 (21.1)	37 (20.7)	7 (23.3)	0.74
Smoking, n (%)	99 (47.4)	84 (46.9)	15 (50)	0.13
Family history, n (%)	99 (47.9)	83 (46.4)	16 (53.3)	0.48
CAD history, n (%)	63 (30.1)	54 (30.2)	9 (29.9)	0.96
High Killip class, n (%)	10 (4.8)	7 (3.9)	3 (10)	0.15
LV ejection fraction, %	49.7 ± 7.3	50.1 ± 7.2	47.0 ± 7.3	0.03
Medications, n (%)				
Acetylsalicylic acid	83 (39.7)	73 (40.8)	10 (33.3)	0.44
P_2_Y_12_ blockers	10 (4.8)	10 (5.6)	0 (0)	0.19
Beta-blockers	58 (27.2)	54 (30.2)	4 (13.3)	0.06
ACEI	62 (29.7)	55 (30.7)	7 (23.3)	0.41
ARB	29 (13.9)	25 (14)	4 (13.3)	0.93
Statins	25 (12)	23 (12.8)	2 (6.7)	0.33
GRACE risk score	97 ± 26	97 ± 25	100 ± 27	0.45
In-hospital mortality, n (%)	5 (2.4)	3 (1.7)	2 (6.7)	0.41
CV-caused hospitalization, n (%)	6 (2.9)	4 (2.2)	2 (6.7)	0.18

Abbreviations: NR, no-reflow; BMI, body mass index; CAD, coronary artery disease; LV, left ventricular; CV, cardiovascular; ACEI, angiotensin converting enzyme inhibitor; ARB, angiotensin receptor blocker; GRACE, Global Registry risk score for Acute Coronary Events.

Table 2 illustrates the laboratory and angiographic features of all recruited patients. Lipid profiles and fasting blood glucose values were not significantly different between the groups. CRP and CAR levels were higher, whereas albumin levels were lower in individuals who acquired NR. The presence of high thrombus burden was substantially greater in the NR group, with 52 (24.9%) individuals having a high thrombus burden. The frequency of post-TIMI flow 0-2 was higher in the NR group, as predicted. In patients who had NR and those who did not, both SYNTAX score I and SYNTAX score II were comparable.

**Table 2:** Laboratory and angiographic characteristics of the study population

	**All patients** **(n=209)**	**NR (-)** **(n=179)**	**NR (+)** **(n=30)**	***P*** ** value**
eGFR, mL/min/1.73 m^2^	89 ± 22	88 ± 22	95 ± 18	0.10
Fasting glucose, mg/dL	122 [104-167]	123 [106-170]	121 [92-151]	0.74
TC, mg/dL	204 ± 53	201 ± 55	222 ± 43	0.06
LDL-C, mg/dL	125 ± 46	122 ± 46	142 ± 38	0.02
HDL-C, mg/dL	39 ± 9.4	39 ± 9.5	40 ± 8.8	0.70
Triglyceride, mg/dL	168 [117-247]	165 [113-246]	181 [154-285]	0.36
Neutrophil, µx10^3^ /µL	6.2 [4.6-8.1]	6.1 [4.6-8.2]	7.0 [5.2-8.1]	0.62
Lymphocyte, µx10^3^ /µL	2.4 [1.9-3.2]	2.4 [1.9-3.2]	2.6 [2.1-3.1]	0.50
Platelet, µx10^3^ /µL	234 ± 68	231 ± 69	249 ± 62	0.19
CRP, mg/dL	5.8 [3.1-10.3]	5.7 [3.1-10]	6.8 [4.0-16.5]	0.009
Albumin, g/d	3.9 [3.6-4.2]	3.9 [3.7-4.2]	3.8 [3.4-4.0]	0.01
CAR	1.5 [0.8-2.8]	1.4 [0.8-2.7]	1.7 [1.0-4.3]	0.001
High thrombus burden, n (%)	52 (24.9)	38 (21.2)	14 (46.7)	0.003
Post-procedural TIMI flow				
TIMI 0	4 (1.9)	0 (0)	4 (13.3)	
TIMI 1	10 (4.8)	0 (0)	10 (33.3)	< 0.01
TIMI 2	16 (7.7)	0 (0)	16 (53.3)	
TIMI 3	179 (85.6)	179 (100)	0 (0)	
SYNTAX score I	12 [7- 24]	12 [7- 24]	16 [9- 23]	0.52
SYNTAX score II	23 [18- 29]	24 [18- 30]	21 [17- 27]	0.34

Abbreviations: NR, no-reflow; eGFR, estimated glomerular filtration rate; TC, total cholesterol, LDL-C, low-density lipoprotein-cholesterol, HDL-C, high-density lipoprotein-cholesterol; CAR, C-reactive protein to albumin ratio; TIMI, thrombolysis in myocardial infarction.

 We conducted both univariate and multivariate regression methods to determine the predictors of NR (Table 3). In univariate logistic regression model, the CAR, albumin, CRP, left ventricular (LV) ejection fraction, LDL-C, and high thrombus grade were all found to be predictors of NR. CAR (OR: 1.250, 95 %CI: 1.033-1.513, *P* = 0.02), elevated LDL-C level, and high thrombus grade were all found to be independent predictors of NR in multivariate regression model. According to our multivariate regression model, albumin and CRP, on the other hand, were not demonstrated to be associated with the NR formation.

**Table 3:** Factors that were found to be independently associated with the no-reflow in univariate and multivariate logistic regression analysis

	**Univariate**		Multivariate*	
	**OR (95% CI)**	***P*** ** value**	**OR (95% CI)**	***P*** ** value**
CAR	1.312 (1.099-1.566)	< 0.01	1.250 (1.033-1.513)	0.02
CRP	1.064 (1.014-1.11)	0.01	1.044 (0.989-1.102)	0.11
Albumin	0.303 (0.115-0.797)	0.02	0.454 (0.165-1.248)	0.13
LV ejection fraction	0.944 (0.896-0.995)	0.03	0.967 (0.914-1.023)	0.25
LDL-C	1.011 (1.001-1.020)	0.03	1.012 (1.002-1.022)	0.02
Thrombus grade ≥ 4	3.247 (1.456-7.238)	< 0.01	2.863 (1.227- 6.684)	0.01

Abbreviations: CAR, C-reactive protein to albumin ratio; CRP, C-reactive protein; LV, left ventricular; LDL-C, low-density lipoprotein-cholesterol. *The variables with a p-value of less than 0.1 in the univariate analysis were incorporated into the multivariate logistic regression analysis by using enter method.

**Figure 1:**
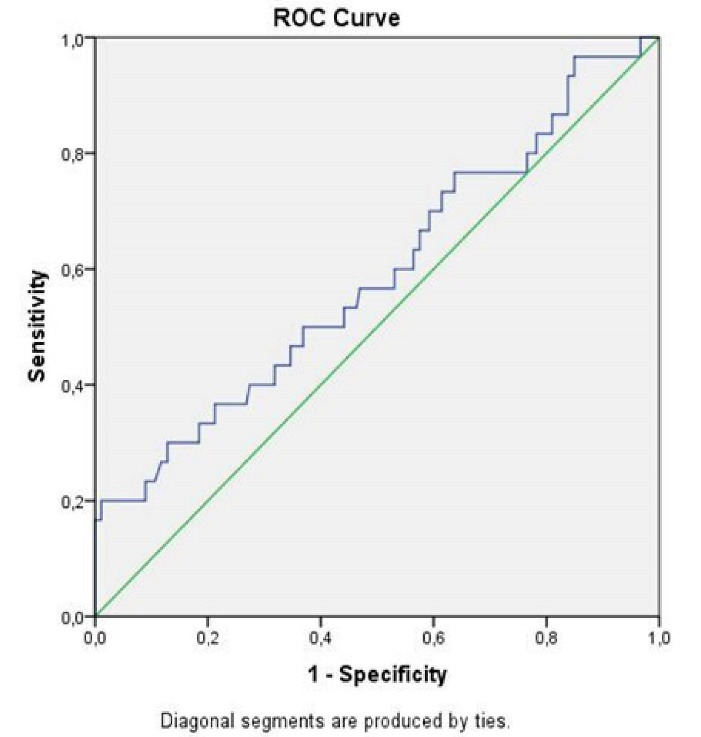
A ROC curve analysis for detecting the development of no-reflow phenomenon in non-STEMI patients.

 The optimal level for CAR in detecting NR was revealed to be > 1.4 in a ROC curve assessment, with 60% sensitivity and 47% specificity (area under curve: 0.59, 95%CI: 0.47-0.71, *P*< 0.01). (Figure 1).

## Discussion

 Our data showed that the CAR was linked to the NR phenomenon in patients with non-STEMI after PCI. To our knowledge, this is the first study showing a link between the CAR and NR phenomena in non-STEMI subjects.

 PCI is the standard therapy for non-STEMI patients to restore an epicardial blood flow in an infarct-related artery (IRA), particularly in those with having high-risk features. Even though the restoring of a normal epicardial flow in the IRA, the NR phenomenon occurs in 15-40% of non-STEMI patients. In our investigation, the incidence of the NR phenomenon after PCI was 6.9% across all non-STEMI patients, which was consistent with the previous results in the literature.^1,2,17^ The actual mechanisms for the occurrence of NR are yet unknown; however, inflammation appears to be one of the most likely contributor. Researchers have revealed that elevated CRP, platelet count, and white blood cell counts, particularly neutrophils and lymphocytes, are related with the NR phenomenon.^18,19^

 Albumin, as a negative acute phase protein and a nutritional marker, has an influence on the development of atherosclerosis and unfavorable cardiovascular events, such as NR.^9^ Further, hypoalbuminemia was found to be a predictor of poor outcomes in patients with ACS by Polat et al.^20^ Additionally, lower levels of albumin on admission, even in the normal range, were a predictor of death in ACS individuals.^21^ In contrast to these findings, our multivariate model found that neither CRP nor albumin were associated to the formation NR in this investigation.

 The CAR, a newly established inflammatory index, may properly represent the systemic inflammatory process and has been related with poor prognosis in other noncardiac clinical situations when compared to these two indicators independently.^8,9,22^ This inflammatory index’s prognostic value in patients with STEMI and non-STEMI had also been explored. The CAR was found to be a predictor of coronary artery disease severity in individuals with non-STEMI by Kalyoncuoglu et al^23^ CAR was found to be a predictor of poor outcomes in STEMI patients by Çınar et al.^12^ To our knowledge, the prognostic power of CAR in predicting NR in non-STEMI patients having PCI has not been investigated. The CAR was found to be greater in patients with the NR phenomenon according to the findings of this study. Furthermore, when compared to other markers, such as CRP and albumin, only CAR was found to be independently linked with NR occurrence.

 We consider that our findings are useful in clinical settings. In non-STEMI patients undergoing PCI, the CAR can be a valuable inflammatory biomarker for identifying high-risk individuals for NR. CAR can also be used to identify high-risk subgroups since it is a simple, affordable, and easily available index. However, because this was a retrospective investigation with a small number of cases, large and prospective studies are needed to clarify the accuracy of CAR in predicting the development of the NR phenomenon and determining its diagnostic value, particularly in non-STEMI individuals.

 Our research has certain limitations. Initially, the investigation was carried out retrospectively with a small number of patients. Second, because of the small number of participants, the prognostic significance of CAR could not be assessed in this study. Third, while the study included only consecutive non-STEMI patients, we acknowledged the possibility of selection bias. Fourth, only visual evaluation was used to quantify NR. As a consequence, studies adopting more advanced imaging modalities are required to assess NR and support our findings. Fifth, we acknowledge the lack of data regarding the time delay to CAG and intervention in non-STEMI patients. Finally, we did not collect the data regarding the long-term outcomes in non-STEMI patients with and without NR.

## Conclusion

 In this research, we revealed that the CAR was independently related with NR in non-STEMI patients having PCI.

## Author Contributions

**Conceptualization: **Aydın Rodi Tosu, Tufan Çınar, Muhsin Kalyoncuoğlu.

**Methodology: **Aydın Rodi Tosu, Tufan Çınar, Muhsin Kalyoncuoğlu, Halil İbrahim Biter, Sinem Çakal, Beytullah Çakal.

**Validation: **Aydın Rodi Tosu, Tufan Çınar, Muhsin Kalyoncuoğlu.

**Formal Analysis:** Muhsin Kalyoncuoğlu.

**Investigation: **Murat Selçuk, Erdal Belen, Mehmet Mustafa Can.

**Resources: **Halil İbrahim Biter, Sinem Çakal, Beytullah Çakal.

**Data Curation:** Aydın Rodi Tosu, Halil İbrahim Biter, Sinem Çakal.

**Writing—Original Draft Preparation: **Aydın Rodi Tosu, Tufan Çınar.

**Writing—Review and Editing: **Aydın Rodi Tosu, Tufan Çınar, Murat Selçuk, Erdal Belen, Mehmet Mustafa Can.

**Visualization:** Halil İbrahim Biter, Sinem Çakal, Beytullah Çakal, Murat Selçuk, Erdal Belen, Mehmet Mustafa Can.

**Supervision: **Erdal Belen, Mehmet Mustafa Can.

**Project Administration:** Aydın Rodi Tosu, Tufan Çınar, Muhsin Kalyoncuoğlu, Halil İbrahim Biter.

**Funding Acquisition:** Aydın Rodi Tosu, Tufan Çınar.

## Ethical Approval

 The study was approved by the Institute Review Board (decision number: 705). The study was carried out in accordance with recommendations of the Declaration of Helsinki.

## Competing Interests

 The authors declare that they have no competing interests.

## Funding

 None.

## References

[1] Jaffe R, Charron T, Puley G, Dick A, Strauss BH (2008). Microvascular obstruction and the no-reflow phenomenon after percutaneous coronary intervention. Circulation.

[2] Wang L, Cheng Z, Gu Y, Peng D (2015). Short-term effects of verapamil and diltiazem in the treatment of no reflow phenomenon: a meta-analysis of randomized controlled trials. Biomed Res Int.

[3] Niccoli G, Rigattieri S, De Vita MR, Valgimigli M, Corvo P, Fabbiocchi F (2013). Open-label, randomized, placebo-controlled evaluation of intracoronary adenosine or nitroprusside after thrombus aspiration during primary percutaneous coronary intervention for the prevention of microvascular obstruction in acute myocardial infarction: the REOPEN-AMI study (Intracoronary Nitroprusside Versus Adenosine in Acute Myocardial Infarction). JACC Cardiovasc Interv.

[4] Prati F, Romagnoli E, Limbruno U, Pawlowski T, Fedele S, Gatto L (2015). Randomized evaluation of intralesion versus intracoronary abciximab and aspiration thrombectomy in patients with ST-elevation myocardial infarction: the COCTAIL II trial. Am Heart J.

[5] Sakakura K, Funayama H, Taniguchi Y, Tsurumaki Y, Yamamoto K, Matsumoto M (2017). The incidence of slow flow after rotational atherectomy of calcified coronary arteries: a randomized study of low speed versus high speed. Catheter Cardiovasc Interv.

[6] Bekkers SC, Yazdani SK, Virmani R, Waltenberger J (2010). Microvascular obstruction: underlying pathophysiology and clinical diagnosis. J Am Coll Cardiol.

[7] Fröhlich GM, Meier P, White SK, Yellon DM, Hausenloy DJ (2013). Myocardial reperfusion injury: looking beyond primary PCI. Eur Heart J.

[8] Kinoshita A, Onoda H, Imai N, Iwaku A, Oishi M, Tanaka K (2015). The C-reactive protein/albumin ratio, a novel inflammation-based prognostic score, predicts outcomes in patients with hepatocellular carcinoma. Ann Surg Oncol.

[9] Fairclough E, Cairns E, Hamilton J, Kelly C (2009). Evaluation of a modified early warning system for acute medical admissions and comparison with C-reactive protein/albumin ratio as a predictor of patient outcome. Clin Med (Lond).

[10] Karabağ Y, Çağdaş M, Rencuzogullari I, Karakoyun S, Artaç İ, İliş D (2018). Usefulness of the C-reactive protein/albumin ratio for predicting no-reflow in ST-elevation myocardial infarction treated with primary percutaneous coronary intervention. Eur J Clin Invest.

[11] Karabağ Y, Çağdaş M, Rencuzogullari I, Karakoyun S, Artaç İ, İliş D (2019). The C-reactive protein to albumin ratio predicts acute kidney injury in patients with ST-segment elevation myocardial infarction undergoing primary percutaneous coronary intervention. Heart Lung Circ.

[12] Çınar T, Çağdaş M, Rencüzoğulları İ, Karakoyun S, Karabağ Y, Yesin M (2019). Prognostic efficacy of C-reactive protein/albumin ratio in ST elevation myocardial infarction. Scand Cardiovasc J.

[13] Amstardam EA, Wenger NK, Brindis RG, Donald E, CaseyJr CaseyJr, Theodore G (2014). Ganiats, et al 2014 AHA/ACC Guideline for the management of patients with non–ST-elevation acute coronary syndromes: a report of the American College of Cardiology/American Heart Association Task Force on Practice Guidelines. JACC.

[14] Çağdaş M, Rencüzoğullari I, Karakoyun S, Karabağ Y, Yesin M, Artaç I (2019). Assessment of relationship between C-reactive protein to albumin ratio and coronary artery disease severity in patients with acute coronary syndrome. Angiology.

[15] Gibson CM, de Lemos JA, Murphy SA, Marble SJ, McCabe CH, Cannon CP (2001). Combination therapy with abciximab reduces angiographically evident thrombus in acute myocardial infarction: a TIMI 14 substudy. Circulation.

[16] Kurtul A, Yarlioglues M, Celik IE, Duran M, Elcik D, Kilic A (2015). Association of lymphocyte-to-monocyte ratio with the no-reflow phenomenon in patients who underwent a primary percutaneous coronary intervention for ST-elevation myocardial infarction. Coron Artery Dis.

[17] Niccoli G, Burzotta F, Galiuto L, Crea F (2009). Myocardial no-reflow in humans. J Am Coll Cardiol.

[18] Celik T, Balta S, Mikhailidis DP, Ozturk C, Aydin I, Tok D (2017). The relation between no-reflow phenomenon and complete blood count parameters. Angiology.

[19] Ndrepepa G, Tiroch K, Keta D, Fusaro M, Seyfarth M, Pache J (2010). Predictive factors and impact of no reflow after primary percutaneous coronary intervention in patients with acute myocardial infarction. Circ Cardiovasc Interv.

[20] Polat N, Oylumlu M, Işik MA, Arslan B, Özbek M, Demir M (2020). Prognostic significance of serum albumin in patients with acute coronary syndrome. Angiology.

[21] Plakht Y, Gilutz H, Shiyovich A (2016). Decreased admission serum albumin level is an independent predictor of long-term mortality in hospital survivors of acute myocardial infarction. Soroka Acute Myocardial Infarction II (SAMI-II) project. Int J Cardiol.

[22] Ranzani OT, Zampieri FG, Forte DN, Azevedo LC, Park M (2013). C-reactive protein/albumin ratio predicts 90-day mortality of septic patients. PLoS One.

[23] Kalyoncuoglu M, Durmus G (2020). Relationship between C-reactive protein-to-albumin ratio and the extent of coronary artery disease in patients with non-ST-elevated myocardial infarction. Coron Artery Dis.

